# Distribution of ABO and rhesus blood grouping with HIV infection among blood donors in Ekiti State Nigeria

**DOI:** 10.1097/MD.0000000000036342

**Published:** 2023-11-24

**Authors:** Arinze Favour Anyiam, Onyinye Cecilia Arinze-Anyiam, Emmanuel Anyachukwu Irondi, Emmanuel Ifeanyi Obeagu

**Affiliations:** a Department of Medical Laboratory Science, Haematology Unit, Faculty of Basic Medical and Health Sciences, Thomas Adewumi University, Oko, Nigeria; b Department of Medical Laboratory Science, Haematology Unit, Faculty of Basic Medical and Health Sciences, Thomas Adewumi University, Oko, Nigeria; c Department of Medical Biochemistry and Pharmacology, Faculty of Pure and Applied Sciences, Kwara State University, Malete, Nigeria; d Department of Medical Laboratory Science, Kampala International University, Uganda.

**Keywords:** antigens, blood bank, blood group, erythrocyte, etiological

## Abstract

Erythrocyte antigens, particularly those which give rise to different blood group systems, are potentially known to perform as receptor sites for different types of disease-causing agents. It is for this reason that this study was carried out to determine the distribution of different blood groups and how susceptible they are to human immunodeficiency virus (HIV) infection. For this study, data were retrieved from different blood bank registers at 4 major blood banks in Ekiti State (National Blood Transfusion Services, Ado, State Specialist Hospital, Ikole, State Specialist Hospital, Ijero, State Specialist Hospital, Ikere. All in Ekiti State). Demographic data such as age and gender were collected on 2388 individuals who were recruited at the above stated facilities over a 2-year period. Their blood groups (Rhesus and ABO) and HIV status were equally recorded. Results of the ABO blood group analysis of the subjects showed that Blood Group O had the highest population (78.2%) while blood Group AB had the lowest (0.9%). The percentages of Rhesus positive and negative persons observed in this population were 94.7% and 5.3%, respectively. The total sero-prevalence of HIV infection was 0.81%. However, there was no significant difference in the prevalence of HIV among the different ABO and Rhesus blood types. This study revealed no association between ABO and Rhesus blood groups and HIV infection.

## 1. Introduction

The ABO and Rh (Rhesus) blood groups are the most popular of all the human blood groups.^[1]^ Blood transfusion is a very noble lifesaving practice. However, there are accompanying risks of spreading transfusion transmissible infections (TTIs) through transfusion of unscreened blood. Some of these diseases like ABO and Rh Haemolytic Disease of the Foetus and Newborn, diabetes mellitus and duodenal ulcer, have been shown to be related to a particular blood group, while others are actually transmitted via transfusion.^[2]^ The WHO^[3]^ lamented that just about 66% of low-income countries subject blood donations to routine TTI testing for diseases like hepatitis C virus, hepatitis B virus, human immunodeficiency virus (HIV) and syphilis,^[4]^ unlike high-income countries with a routine TTI screening percentage of 99.6%. It is also estimated that close to 10% of blood donations are deferred because of TTIs.^[4]^ It is important to understand blood groups and how they are associated with different diseases. Several studies have been carried out on the link between the ABO blood group and diseases like HIV, ovarian cancer, malaria, and cerebral thrombosis.^[5–7]^

In Nigeria, few studies have been carried out on the relationship between blood groups and HIV. This paucity of information prompted this research. Every single human blood group system indicates the type of antigens that are present on the RBC surface. Knowledge of the association between red cell surface antigens and diverse chronic diseases is important in seeking better treatments. Although several studies have been carried out to find the association between TTIs like HIV with ABO blood groups, the results have been conflicting, probably because of differences in sample size, screening methods, geographical and social factors. Our study, therefore, was intended on finding out the distribution of HIV infection in different ABO and Rh blood groups among blood donors in Ekiti State, Nigeria.

## 2. Materials and methods

The study was carried out at the National Blood Transfusion Services Ado Ekiti, State Specialist Hospital, Ikole, State Specialist Hospital, Ijero and General Hospital Ikere Ekiti, all located in Ekiti State, Southwestern Nigeria.

A total number of 2338 subjects comprising 1988 male (85.03%) and 350 female (14.97%) retrospective donors were recruited from February 2018 to February 2020, at the following study areas: National Blood Transfusion Service Ado-Ekiti, State Specialist Hospital Ikole, State Specialist Hospital Ijero, State Specialist Hospital Ikere, all located in Ekiti State, Nigeria.

The study was a retrospective and prospective study. A total of 360 prospective blood donors and 1978 retrospective blood donor records were employed for the study. The donors’ records from February 2018 up till February 2020 were obtained and prospective donors who had been donating for the past 2 years were also recruited for the study. Records of donors’ register were statistically analyzed, the donors with more than 1 entry in the record were included once and also 300 prospective donors were also screened to complement the study.

Ethical approval and clearance for this study was obtained from the Ekiti State Hospitals Management Board Ethical Committee in compliance with the ethical principles that originate from the Helsinki declaration. Samples were obtained from donors who were attending the blood banking center (Haematology) in stipulated hospitals. The sample collection process was explained to the donors while their informed consents were sought and obtained prior to commencement of the study. Data obtained included: socio-demographic characteristics (age, education level, occupation, and ethnic group social class) and donation duration.

A total of 4 mL of blood sample were collected from prospective donors through clean venepuncture and 2 mL was dispensed into EDTA (Ethylenediamine tetra-acetic acid) bottles, while the remaining 2 mL was dispensed into plain vacutainer bottles. Samples were transported in a well-insulated box, containing freezer packs and stored at 4^°^C prior to analysis. Blood grouping was carried out on the blood sample collected inside the EDTA bottle by agglutination testing and the plain bottle samples were centrifuged to separate the red cell from the serum. The serum was aspirated and screened for HIV using enzyme-linked immunosorbent assay technique.

For ABO and Rh grouping, these were done using the Standard tube method.^[8]^ The blood samples were mixed with anti-A and anti-B sera directly in labeled tubes. Agglutination of red blood cells after mixing indicated the presence of A or B antigens in either tube, and interpreted as blood group A or B. Absence of agglutination in both tubes was interpreted as blood group O, while agglutination in both tubes was deduced as blood group AB. A negative control tube was used to ensure accuracy, while microscopic examination was used to confirm non-agglutination.

For the HIV screening by enzyme-linked immunosorbent assay,^[9]^ the micro titer plate wells were coated with antigen. All unbound sites were blocked to prevent false positive results. Sample containing antibody were added to the wells and the plate incubated at 37°C. The plates were then washed, so that unbound antibody is removed. Secondary antibody conjugated to an enzyme was added. The plates were washed, so that unbound enzyme-linked antibodies were removed. Substrate which is converted by the enzyme to produce a colored product was added. Then there was reaction of the substrate with the enzyme to produce a colored product.

Statistical analysis was carried out using the IBM SPSS statistics software (version 20, USA). ANOVA was used to test for the significance of differences between 2 groups. Pearson’s correlation coefficient was used to correlate the relationship between different blood types and their correlation with HIV infections. *P* < .05 was considered statistically significant.

## 3. Results

Out of the 2338 subjects, 360 blood donors consisting of 313 males (86.94%) and 47 females (13.06%) were recruited as prospective donors for the study, while the remaining 1978 subjects who were made up of 1675 males (84.68%) and 303 females (15.32%) retrospective donors were recorded from the above-named hospitals. Therefore, a total number of 1988(%) males and 350(%) females were used for the study (Table 1).

**Table 1:** Gender distribution of both recruited and retrospective donors in Ekiti State from February 2018 to February 2020.

Gender	Recruited donors	Retrospective donors	Total
Male	313 (86.94%)	1675 (84.68%)	1988 (85.03%)
Female	47 (13.06%)	303 (15.32%)	350 (14.97%)
Total	360 (100%)	1978 (100%)	2338 (100%)

The ABO, Rhesus (D) blood group distribution among the blood donors in Ekiti State are shown in Table 2. Out of the 2338 donors, a total number of 2215 (94.75%) were Rh D positive, while 123 (5.25%) were Rhesus (Rh) D negative. Among the Rh D negative subjects, 12 (0.51%) were of blood group A, 19 (0.81%) blood group B, 0 (0.00%) blood group AB, and 92 (3.93%) blood group O. Out of the 2215 (94.75%) Rh D positive subjects, 234 (10.01%) were blood group A, 212 (9.07%) blood group B, 20 (0.86%) blood group AB and 1749 (74.81%) blood group O.

**Table 2:** The ABO Rhesus “D” blood group distribution among blood donors in Ekiti State.

ABO blood group	Rhesus “D” positive (%)	Rhesus “D” negative (%)
A	234 (10.01)	12 (0.51)
B	212 (9.07)	19 (0.81)
AB	20 (0.86)	0 (0.00)
O	1749 (74.81)	92 (3.93)
Total	2215 (94.75)	123 (5.25)

The HIV sero-prevalence among the blood donors in Ekiti State are shown in Table 3. Out of the 2338 donors, Blood group O had the highest number of HIV positive individuals with 13 subjects (0.56%), followed by blood group A with 6 subjects (0.26%), while blood groups B and AB blood groups had the least number of 0 subjects (0.00%), respectively. On the other hand, blood group O had the highest number of HIV negative subjects with 1830 subjects (78.83%), followed by blood group A with 240 subjects (10.27%), while blood group B had 229 subjects (9.79%) and blood group AB had the least number of 20 subjects (0.86%), respectively.

**Table 3:** The HIV sero-prevalence among donor groups in Ekiti State.

ABO blood group	HIV positive (n)	Prevalence (%)
A	6	0.26
B	0	0.00
AB	0	0.00
O	13	0.56
Total	19	0.81

Table 4 below shows the HIV prevalence according to ABO blood group donors. The percentage of infected individuals was highest in blood group A (2.44%) followed by blood group O (0.71%). There were no observed HIV infected blood donors among group B (0.00%) and group AB (0.00%) throughout the period of study.

**Table 4:** HIV prevalence according to ABO blood group donors.

Total donors	Blood group A N = 246 (10.52%)		Blood group B N = 229 (9.79%)		Blood group AB N = 20 (0.86%)		Blood group O N = 1843 (78.83%)	
	HIV+ve	HIV-ve	HIV+ve	HIV-ve	HIV+ve	HIV-ve	HIV+ve	HIV-ve
	1 (1.89%)	52	0 (0.00%)	39	0 (0.00%)	3	4 (0.79%)	1 (1.89%)
	3 (2.59%)	113	0 (0.00%)	92	0 (0.00%)	8	2 (0.34%)	3 (2.59%)
	2 (2.30%)	75	0 (0.00%)	98	0 (0.00%)	9	7 (0.94%)	2 (2.30%)
2338	6	240	0	229	0	20	13	1830

For the distribution of HIV prevalence according to year of blood donation (Table 5), there were a total of 602 donors in 2018, comprising 5 HIV-positive individuals (0.83%) and 597 HIV-negative subjects (99.17%), while in 2019, there were a total of 810 donors comprising 5 HIV-positive individuals (0.62%) and 805 HIV-negative persons (99.38%). In 2020, 926 donors were captured in the study, comprising 9 HIV-positive subjects (0.97%) and 917 HIV-negative persons (99.03%) and were least infected in the year 2018 (1.89%). However, among blood group O donors, the highest HIV infectivity was recorded in the year 2020 (0.94%), followed by 2018 (0.79%) and least was recorded in 2019 (0.34%). Also, out of the total population of 2338 donors, blood group O had 1843 donors (78.74%) followed by blood group A with 246 donors (10.52%), blood group B with 231 donors (9.88%) and blood group AB with 20 (0.86%) donors.

**Table 5:** Distribution of HIV prevalence according to year of blood donation.

Year	Total donors	HIV+ve	HIV-ve
2018	602	5 (0.83%)	597 (99.17%)
2019	810	5 (0.62%)	805 (99.38%)
2020	926	9 (0.97%)	917 (99.03%)
Total	2338	19 (0.81%)	2319 (99.19%)

In Table 6 below, it was observed that out of 2215 Rh D positive blood donors, 15 (0.68%) were infected with HIV, while 2200 (99.32%) were HIV negative. While out of 123 Rh (D) negative blood donors, 4 (3.25%) donors were infected with HIV while 119 (96.75%) were HIV negative.

**Table 6:** Percentage of HIV infected individuals within Rh blood system among the blood donors of Ekiti State.

	Rh (D) positive	Rh (D) negative	Total
HIV positive	15 (0.68%)	4 (3.25%)	19
HIV negative	2200 (99.32%)	119 (96.75%)	2319
Total	2215	123	2338

## 4. Discussion

This study presented a higher number of male donors (1988 males, 85.03%) compared to female donors (350 females, 14.97%). This is probably because males have higher red cell and platelet counts than females in most cases,^[10]^ while females donate less often, probably because of the physiological demands of pregnancy and menstruation.^[11]^

Our study showed that blood group O had the highest frequency of 1841 donors (78.74%), followed by blood group A with 246 donors (10.52%), blood group B with 231 donors (9.88%) and blood group AB with 20 (0.86%) donors, respectively. This is in agreement with the study by Siransy et al^[12]^ who reported a high frequency of blood donors in Côte d’Ivoire as group O (49.74%), and least as group AB (4.40%), respectively. The reason for this may be because blood group O individuals are regarded as universal donors due to the absence of A and B antigens on their red cell surfaces. This report showed that more persons donate blood especially group O individuals compared to other parts of the world where such research has been conducted. This is a good step in saving life and the level of awareness should be maintained to ensure regular supply of blood to the required patients for improved well-being and life.

In this study, we also found out that blood group O had the highest percentage of HIV infected donors (0.56%) followed by group A (0.26%), while blood groups B and AB had no cases (0.00%, respectively) as shown in Table 3. In the same vein, HIV incidence was reduced among Rh d negative subjects. However, this contradicts with the findings of^[13]^ but agrees with the finding.^[10]^

Our study recorded an HIV seroprevalence of 0.82% which is slightly lower than the seroprevalence of 2.8% recorded in the study carried out by Hassan et al^[14]^ in Kaduna State, Nigeria. The slight reduction in our study could be as a result of an improvement in HIV/AIDS awareness campaigns by Governmental and non-governmental Organizations in Ekiti State and also meticulous selection undertaken during the donor selection process to remove any blood donors with risk factors capable of causing infection. It may be possible that individuals of blood groups B and AB have some form of increased natural resistance to HIV.^[10]^

## 5. Conclusions

The results of the present study indicate that HIV is strongly associated with Rh positive blood groups. Blood group O Rh D positive phenotype showed the highest seroprevalence for HIV among blood donors. There was no significant association of HIV with any specific ABO and Rh blood group but rather a reduced seropositivity for HIV in Rh negative blood groups. Therefore, more studies with a bigger sample size and performed over a longer duration of time are needed to study any association between blood group and HIV.

## Acknowledgments

The authors acknowledge the Ekiti State Hospitals Management Board Ado-Ekiti for the technical assistance provided for this study.

## Author contributions

**Conceptualization:** Arinze Favour Anyiam, Onyinye Cecilia Arinze-Anyiam, Emmanuel Anyachukwu Irondi, Emmanuel Ifeanyi Obeagu.

**Data curation:** Arinze Favour Anyiam.

**Formal analysis:** Arinze Favour Anyiam.

**Investigation:** Arinze Favour Anyiam, Onyinye Cecilia Arinze-Anyiam, Emmanuel Anyachukwu Irondi, Emmanuel Ifeanyi Obeagu.

**Methodology:** Arinze Favour Anyiam, Onyinye Cecilia Arinze-Anyiam, Emmanuel Anyachukwu Irondi, Emmanuel Ifeanyi Obeagu.

**Supervision:** Emmanuel Ifeanyi Obeagu.

**Validation:** Arinze Favour Anyiam, Emmanuel Ifeanyi Obeagu.

**Visualization:** Emmanuel Ifeanyi Obeagu.

**Writing – original draft:** Arinze Favour Anyiam, Onyinye Cecilia Arinze-Anyiam, Emmanuel Anyachukwu Irondi, Emmanuel Ifeanyi Obeagu.

**Writing – review & editing:** Onyinye Cecilia Arinze-Anyiam, Emmanuel Anyachukwu Irondi, Emmanuel Ifeanyi Obeagu.
